# Radial-specific catheters for neuroendovascular procedures: A systematic review and meta-analysis

**DOI:** 10.1007/s10143-025-03765-x

**Published:** 2025-08-28

**Authors:** Ali Mortezaei, Nadir Al-Saidi, Ibrahim Mohammadzadeh, Jamal Behnood, Muhammed Amir Essibayi, Khaled M. Taghlabi, Ahmed Abdelsalam, Mohammad Amin Habibi, Bardia Hajikarimloo, Luis Guada-Delgado, Ram Saha, Redi Rahmani, Amir H. Faraji, Robert M. Starke

**Affiliations:** 1 Student Research Committee, Gonabad University of Medical Sciences, Gonabad, Iran; 2 College of Medicine, Central Michigan University, Mount Pleasant, USA; 3 Skull Base Research Center, Loghman-Hakim Hospital, Shahid Beheshti University of Medical Sciences, Tehran, Iran; 4 Department of Neuroscience, Addiction Studies, and Neuroimaging, School of Advanced Technologies in Medicine, Tehran University of Medical Sciences, Tehran, Iran; 5 Montefiore-Einstein Cerebrovascular Research Lab, Montefiore Medical Center, Albert Einstein College of Medicine, Bronx, NY USA; 6 Department of Neurological Surgery, Montefiore Medical Center, Albert Einstein College of Medicine, Bronx, NY USA; 7 Clinical Innovations Laboratory, Houston Methodist Research Institute, Houston, TX USA; 8 Department of Neurological Surgery, Radiology, Neurosciences, Pharmacology, University of Miami School of Medicine, Miami, FL USA; 9 Department of Neurosurgery, Shariati Hospital, Tehran University of Medical Sciences, Tehran, Iran; 10 Department of Neurological Surgery, University of Virginia, Charlottesville, USA; 11 Virginia Commonwealth University Health System, Richmond, VA USA; 12 Department of Neurosurgery, Perelman School of Medicine, University of Pennsylvania, Philadelphia, PA USA

**Keywords:** Radial-specific catheters, Neurointervention, Armadillo, RIST, Zoom RDL, BMX 81

## Abstract

Despite a radial-first approach in many neurointerventions, there are no systematic reviews and meta-analysis which comprehensively assess radial-specific catheter for neuroendovascular procedures. A systematic literature search was conducted through four electronic databases based on PRISMA 2020 guideline. Risk of bias was assessed employing Risk of Bias in Non-randomized Studies of Interventions (ROBINS-I) tool. A total of eleven studies with 990 patients using Armadillo, RIST, Zoom RDL, and BMX 81 catheters were included. The Armadillo showed significantly lower failure to catheterize the target vessel (0.0% vs. 3.04%, *P* = 0.036) than RIST. The Zoom RDL catheter had relatively higher failure rate of 10.3%. There was no significant difference between the Armadillo and RIST catheters in procedure-related complications. There were no reported cases of arterial spasm or hemorrhage for Armadillo catheter. RIST catheter had a 3.2% rate of neurological complications, a 3.1% rate of transfemoral conversion, and a 1.8% rate of hematoma. The BMX 81 catheter had a 2.5% rate of arterial vasospasm and a 5% rate of procedure-related complications. The Zoom RDL catheter had consistent rates of procedure-related, transfemoral conversion, and neurological complications, all at 6.9%, with insufficient data on other complications.

## Introduction 

Transradial access for neuroendovascular procedures has emerged as an alternative to femoral artery access in the past two decades because of the procedure’s association with lower complication rates, decreased hospital stay, and enhanced patient comfort [1–4]. However, the procedure requires increased skill to navigate the radial artery’s smaller, more tortuous pathways [1, 5]. On the other hand, with the widespread adoption of transradial access in the neurointerventions, a variety of radial-specific catheters have emerged that differ in structural design and performance, warranting evaluation to guide clinical decision-making and optimal outcomes.

This systematic review and meta-analysis combined data from studies evaluating all available radial-specific catheters for neurointerventional procedures, namely the Armadillo, RIST, BMX 81, and Zoom RDL, each providing unique pathways to transradial access complexities. The Armadillo system is known for its dual-mode design, which offers a firmer support mode for greater stability at the target site and a more flexible tracking mode to maneuver around the winding radial artery [6]. The RIST and Ballast systems combine these features with a catheter with a more rigid proximal shaft and a soft distal tip, increasing overall stability but limiting adaptability to complex tortuosities [7]. The BMX 81 system has a larger inner diameter, or bore, reducing flexibility but allowing the catheter to accommodate a wider range of tools or devices for passing through during the procedure [8]. Finally, the Zoom RDL system has a tapered distal tip and a smooth body to balance support and flexibility for a wide range of procedures, although the tip is prone to damage with frequent usage [9].

Using safety and efficacy data, and accounting for aforementioned differences in catheter characteristics, we aim to highlight the promise of these models while identifying areas for future improvement in catheter technology to enhance the practice of transradial access for the field of neuroendovascular intervention.

## Methods

### Eligibility criteria

The inclusion criteria were (1) randomized trials or observational studies, (2) studies that utilized radial-specific catheters for neuroendovascular procedures, (3) adult patients aged ≥ 18 years, and (4) at least one outcome of interest was reported (e.g., successful catheterization, complications, clinical and radiological outcomes). The exclusion criteria consisted of (1) case reports, case series, reviews, and editorials and (2) animal or in-vitro studies.

### Search strategy and selection process

We conducted a comprehensive literature search adhered to Preferred Reporting Items for Systematic Reviews and Meta-Analyses (PRISMA) guidelines. We searched PubMed/MEDLINE, Scopus, and Web of Science up to December 29, 2024. The search strategy encompasses terms such as *“radial specific catheter*,*” “Armadillo*,*” “RIST*,* “ “BMX 81*,*” “Zoom RDL*,*” “trans-radial intervention*,*” “Neurointervention*,*” “and “trans-radial catheter”* to find relevant studies. Additionally, a manual review of the reference lists from the included studies was performed to identify any relevant studies that might have been overlooked during the automated search. Moreover, studies with overlapping patients were excluded. Neurological complications were defined as any new or worsening neurological event occurring after the procedure, including stroke, transient ischemic attack, intracranial hemorrhage, seizures, altered mental status, cranial nerve deficits, or new focal neurological deficits.

### Screening process 

Two independent authors performed the title, abstract, and full-text screening stages, employing pre-established eligibility criteria. A third party resolved any decision-making conflicts.

### Data extraction

Two independent authors extracted data utilizing a standardized Excel sheet, and a third author meticulously reviewed the collected data. Any conflicts were resolved through team consensus.

### Quality and risk of bias assessment

The quality of the included studies was evaluated utilizing the Risk of Bias in Non-randomized Studies of Interventions (ROBINS-I) tool. This tool assesses bias across seven domains: bias due to confounding, selection of participants into the study, classification of interventions, deviations from intended interventions, missing data, measurement of outcomes, and selection of the reported result.

### Statistical analysis

We used the “netmeta” package on R version 4.3.0 for meta-analysis. Proportions and 95% confidence intervals (CIs) were calculated for each study using a random-effects model due to expected institutional and methodological disparity. Additionally, binary outcomes were analyzed using odds ratio (OR) and 95% CIs. We converted the median and range to mean and SD using the proposed method by Luo et al. 9. Statistical heterogeneity was assessed using visual inspection of the forest plots and measured using the I^2^ and chi-square (χ2) tests [10]. All p-values were two-sided, and *P* < 0.05 was considered statistically significant.

## RESULTS

### Study selection

**Fig. 1:**
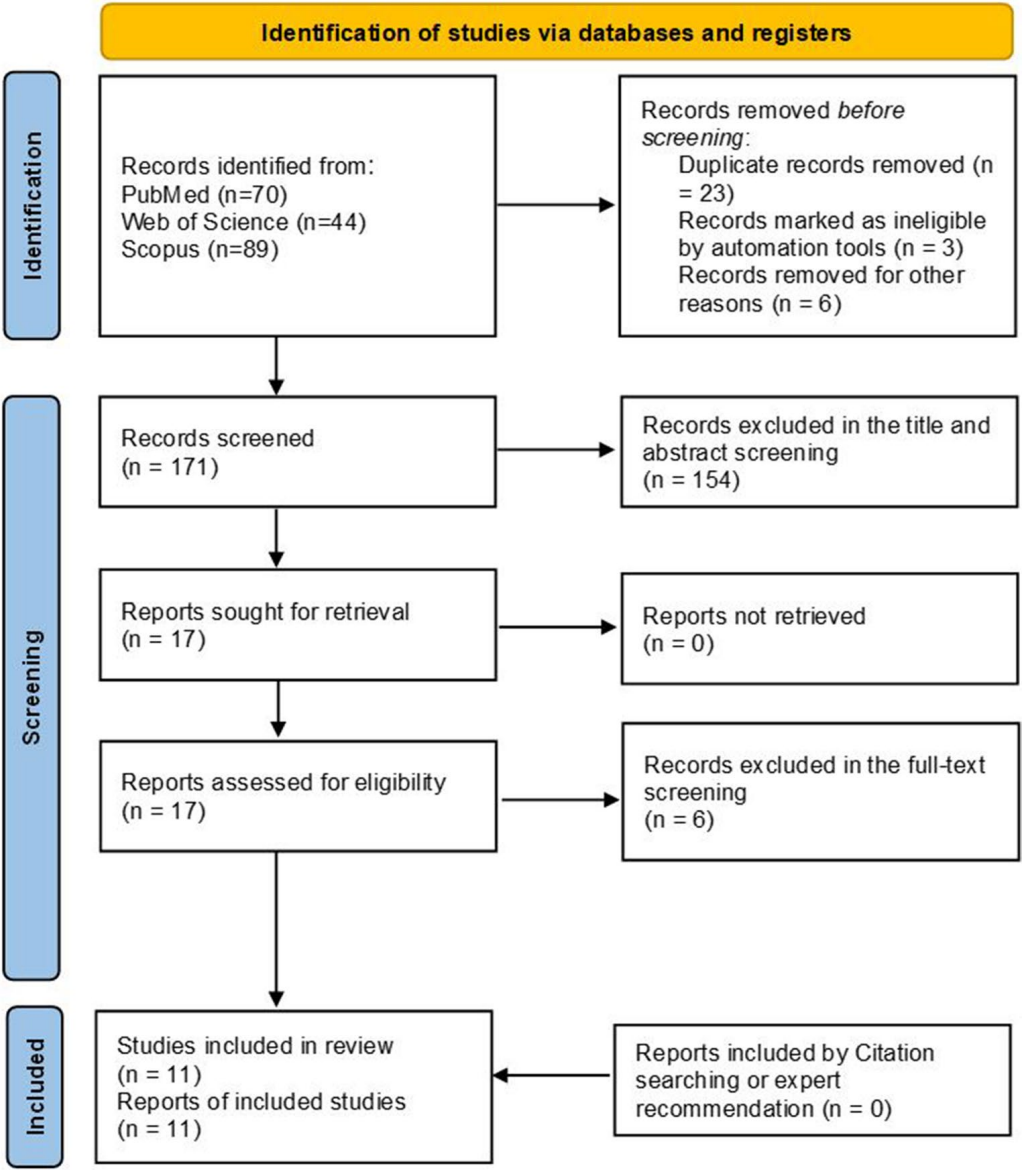
PRISMA flow chart for study selection

Our systematic search yielded 203 studies, of which 35 were left after duplicates were removed. Full-text screening was possible for 18 studies that passed the preliminary title and abstract evaluation. After a careful review, 11 papers [6–9, 11–17] qualified for quantitative synthesis and were included in final examination. Additionally, no additional publications were included after manually checking the listed studies’ referenced sources. The research selection process flowchart is displayed in PRISMA flow diagram in Fig. 1.

>

### Study characteristics

We included 11 studies [6–9, 11–17] with 990 cases in the systematic review and meta-analysis (Table 1). Detailed characteristics of included studies and each catheter and the reason for procedure failure are displayed in Tables 1 and 2. Conversion to femoral access because of anatomical difficulties and procedural failure was the prominent reason for excluding particular cases within studies. Notably, most failure cases were due to the inability to navigate convoluted anatomy or catheterize target vessels (Table 1).

**Table 1:** Characteristics of the included studies

Study	Country	Study Design	Grouping	French	Sample Size	Reason for procedure											Access or procedural failure
						Thrombectomy	Aneurysm treatment	AVM treatment	Carotid artery stent	MMA embolization	AVF treatment	Tumor embolization	Carotid blowout	IA treatment for vasospasm	Epistaxis treatment	Intracranial stent	
El Naamani et al., 2024	USA	Retrospective	Armadillo	7	96	0	47	7	19	9	7	7	0	0	0	0	There was only one case of failure to catheterize the target vessel in each group, with no significant difference between groups (Armadillo: 1.0% vs. RIST: 0.9%, *P* = 0.18). Additionally, both cohorts’ rates of conversion to femoral route were similar (Armadillo: 2.1% vs. RIST: 1.8%, *P* = 0.55).
	USA	Retrospective	RIST	7	110	10	53	6	9	16	7	1	2	3	2	1	
Kappel et al., 2023	USA	Retrospective	Armadillo	7	57	N/A	N/A	1	N/A	1	6	N/A	N/A	N/A	N/A	3	No cases of conversion to a different access route or procedural failure were documented. The Armadillo radial access method was used to successfully perform all procedures with no issues related to catheterization or access failure reported.
Khan et al., 2023	USA	Retrospective	Armadillo	6	9	N/A	9	N/A	N/A	N/A	N/A	N/A	N/A	N/A	N/A	N/A	No instances of procedural failure or conversion to a different access route were documented in this study. The Vecta46 catheter was used to successfully complete all procedures.
Abecassis et al., 2021	USA	Retrospective	RIST	7	152	19	37	4	1	N/A	3	8	N/A	N/A	N/A	N/A	Three cases required conversion to femoral access because the RIST catheter could not be correctly positioned in the left ICA, which was the main cause of procedural failures or conversions. Also, two instances of mechanical thrombectomy necessitated TFA because the aspiration apparatus was difficult to advance. Due to the modest clot size, one case of bilateral subdural hematoma treatment was terminated without any additional intervention after the left ECA could not be accessed.
Allard et al., 2024	France	Retrospective	RIST	7	22	N/A	N/A	N/A	N/A	N/A	N/A	N/A	N/A	22	N/A	N/A	Two instances required a TFA conversion due to inadequate stability with the RIST guide catheter during navigation to the left ICA.
Molinaro et al., 2024	Italy	Retrospective	RIST	N/A	20	N/A	N/A	N/A	N/A	N/A	N/A	N/A	N/A	N/A	N/A	N/A	There were two reported instances of TFA conversion. The first occurred because the acute angle of the brachiocephalic trunk made it difficult to guide the catheter into the carotid artery, and the second occurred because the radial artery in the antecubital area had a loop that made catheter passage highly risky.
Nowak et al., 2023	Poland	Retrospective	RIST	7	237	N/A	N/A	34	N/A	N/A	N/A	N/A	N/A	N/A	N/A	N/A	The researchers noted that five patients needed conversion to femoral access, however they did not specifically explain the reasons for procedural failure or conversion to another access route for each individual case.
Rautio et al., 2023	Finland	Retrospective	RIST	7	100	5	N/A	N/A	3	13	N/A	N/A	N/A	N/A	5	4	TFA was utilized in place of TRA when radial artery diameter was less than optimal (less than 1.7 mm). Four patients (4%) subsequently required conversion to femoral access because of instability.
Waqas et al., 2022	USA	Retrospective	RIST	7	78	6	23	8	3	11	N/A	N/A	N/A	4	6	26	Due to anatomical difficulties, such as an abnormal MMA origin (1 case), unfavorable great vessel geometry (3 cases), and proximal tortuosity of the supra-aortic arteries (1 case), procedural failure and TRA conversion were reported in five cases.
Marangoni et al., 2024	Canada	Retrospective	BMX 81	7	80	N/A	N/A	N/A	N/A	N/A	N/A	N/A	N/A	N/A	N/A	N/A	In one instance, the researchers had to switch to a TFA technique because they could not reach the target vessel. In another case, the radial artery loop experienced vasospasm, which required them to access the ulnar artery instead.
Morsi et al., 2024	USA	Retrospective ​	Zoom RDL	6/7	29	9	8	N/A	N/A	N/A	4	3	N/A	1	1	N/A	Two cases involved conversion to TFA. In one, after having trouble advancing the Zoom RDL, a flow diverter-treated aneurysm necessitated a switch from the TRA to TFA. The other, a subdural hematoma case, required the use of an AXS Infinity LS guide catheter through TFA because the Zoom RDL tip was damaged during repeated attempts via the snuffbox approach.

*TFA* transfemoral access,* TRA*, transradial access, *ICA* internal carotid artery, *ECA* external carotid artery, *MMA* middle meningeal artery

**Table 2:** Details and characteristics of the radial-specific catheters

Device	Manufacturer	Features	ID (in/mm)	OD (in/mm)	Lengths (cm)	Success Rates	Malfunctions	Procedure-Related Complications
Armadillo^6^	Q’Apel	0.072-inch ID; Lubricated coating; Flexible design for radial access	0.072/1.83	0.095/2.41	95, 105	98%	Minor tip damage: 3%; Spasm in cervical segment	Minor spasm: 1.5%; Hand perfusion: 2%
BMX 81^8^	Penumbra	0.081-inch ID; High radial access compatibility; Flexible build	0.081/2.06	0.097/2.46	95, 105, 115	96.80%	Difficulty positioning in ICA	Radial spasm: 3%; Neurological: 1.5%
Zoom RDL^9^	Imperative Care	0.088-inch ID; Hydrophilic coating; Reinforced design for radial stability	0.088/2.24	0.110/2.79	103	95–99%	Tip damage during insertion: 5%	Severe spasm: 6%; Damage during transfer
RIST^7,14^	Medtronic	0.079-inch ID; 7Fr radial access; Stiff shaft; Flexible distal end; Hydrophilic coating	0.079/2.01	0.093/2.36	95, 100	96-98.7%	Instability during navigation: 2%	Radial artery occlusion: 1%; Closure: 2%

Data pooled for the BMX 81, and Zoom RDL catheters was insufficient to directly compare and conduct tests for statistical significance for parameters chosen in this meta-analysis. Thus, direct comparison was only calculated when comparing the Armadillo catheter to the RIST catheter. However, all available pooled data is provided in Tables 2, 3, 4, 5 and 6 for relative comparisons. We separated each catheter’s data to enhance clarity and provide a better understanding of their respective performance.

**Table 3:** Baseline characteristics and demographic of the patients based on each guide catheter

Parameter	Armadillo	RIST	BMX 81	Zoom RDL
No of patients	162	719	80	29
Female	107	195	44	6
Age, mean (SD), y	60.15 (11.3)	62.26 (15.48)	68.31 (14)	61.9 (17.2)
Past Medical History				
Coronary artery disease	43	15	8	N/A
Peripheral vascular disease	21	6	N/A	N/A
Diabetes	50	15	9	N/A
Hypertension	71	52	37	N/A

### Patient demographics

The pooled data revealed that different catheters were associated with specific patient demographics. The Armadillo catheter, utilized on 162 patients, had an average age of 60.15 years (Table 3). In contrast, 719 patients with a mean age of 62.26 years were treated using the RIST catheter. Interestingly, the Armadillo system was used in a significantly greater proportion of female patients than the RIST system (69.2% vs. 52.7%, *P* = 0.038) (Table 6). Proportions of patients with a past medical history of either coronary artery disease, peripheral vascular disease, diabetes, or hypertension are also outlined in Table 3 for Armadillo, RIST, Zoom RDL, and BMX 81.

### Procedural characteristics

#### Reason for procedure

The distribution of procedures performed was influenced by the type of catheter deployed (Table 4). Armadillo catheter was least represented in thrombectomy procedures, with no cases documented (0%). Instead, the Armadillo catheter showed its greatest distribution in aneurysm treatment (56/105 cases, 53.3%). Meanwhile, the RIST catheter had the most diverse application, which included aneurysm treatment (113/340 cases, 33.2%), middle meningeal artery (MMA) embolization (40/288 cases, 13.9%), arteriovenous malformation (AVM) treatment (52/577 cases, 9.0%), carotid artery stenting, tumor embolization, and others. For the Zoom RDL catheter, the greatest proportions of cases were thrombectomies (9/29 cases, 31.0%) followed by aneurysm treatment (8/29 cases, 27.6%). There was insufficient data available to determine the distribution of procedures for the BMX 81 catheter. No significant differences were observed in the number of aneurysm treatments, AVM treatments, MMA embolizations, arteriovenous fistula (AVF) treatments, and intracranial stenting procedures between the Armadillo and RIST catheter systems (Table 6).

**Table 4:** Pooled data for procedure details

Variable	Armadillo	RIST	BMX 81	Zoom RDL
Reason for procedure				
Thrombectomy	0/96 (0%)	40/440 (9.1%)	N/A	9/29 (31.0%)
Aneurysm treatment	56/105 (53.3%)	113/340 (33.2%)	N/A	8/29 (27.6%)
AVM treatment	8/153 (5.2%)	52/577 (9.0%)	N/A	N/A
Carotid artery stent	19/96 (19.8%)	16/440 (3.6%)	N/A	N/A
MMA embolization	10/153 (6.5%)	40/288 (13.9%)	N/A	N/A
AVF treatment	13/153 (8.5%)	10/262 (3.8%)	N/A	4/29 (13.8%)
Tumor embolization	7/96 (7.3%)	9/262 (3.4%)	N/A	3/29 (10.3%)
Carotid blowout	0/96 (0%)	2/110 (1.8%)	N/A	
IA treatment for vasospasm	0/96 (0%)	29/210 (13.8%)	N/A	1/29 (3.4%)
Epistaxis treatment	0/96 (0%)	13/288 (4.5%)	N/A	1/29 (3.4%)
Intracranial stent	3/153 (2.0%)	31/288 (10.8%)	N/A	N/A
Endovascular modality				
Coiling	11/96 (11.5%)	107/621 (17.2%)	2/80 (2.5%)	N/A
Stent-assisted coiling	8/153 (5.2%)	70/469 (14.9%)	3/80 (3.8%)	N/A
Balloon-assisted coiling	1/96 (1.0%)	3/262 (1.1%)	N/A	1/29 (3.4%)
Flow diversion	62/162 (38.3%)	121/462 (2.6%)	16/80 (20%)	5/29 (17.2%)
Embolization	8/153 (5.2%)	111/677 (16.4%)	6/80 (7.5%)	N/A
Contour	3/96 (3.1%)	2/132 (1.5%)	N/A	N/A
WEB	13/153 (8.5%)	35/384 (9.1%)	4/80 (5%)	N/A
Target vessel				
ICA	6/9 (66.7%)	124/272 (45.6%)	N/A	7/29 (2.4%)
MCA	4/9 (44.4%)	29/120 (24.2%)	N/A	18/29 (62.1%)
ACA	N/A	22/100 (22.0%)	N/A	3/29 (10.3%)
AcomA	N/A	3/22 (13.6%)	N/A	1/29 (3.4%)
AICA	N/A	N/A	N/A	1/29 (3.4%)
VA	N/A	18/230 (7.8%)	N/A	N/A
MMA	1/57 (1.8%)	28/178 (15.7%)	N/A	2/29 (6.9%)
Ophthalmic	1/57 (1.8%)	3/78 (3.8%)	N/A	4/29 (13.8%)
PCA	N/A	6/78 (7.7%)	N/A	N/A
PcomA	N/A	7/78 (9.0%)	N/A	N/A
PICA	N/A	1/78 (1.3%)	N/A	N/A
BA	N/A	1/78 (1.3%)	N/A	N/A
SCA	N/A	1/78 (1.3%)	N/A	N/A
Radial laterality				
Right	96/96 (100%)	676/719 (94.0%)	80/80 (100%)	24/29 (82.8%)
Left	0/96 (0%)	56/719 (7.8%)	0/80 (0%)	1/29 (3.4%)
Laterality of pathology				
Right	56/96 (58.3%)	280/382 (73.3%)	N/A	2/29 (6.9%)
Left	39/96 (40.6%)	83/382 (21.7%)	N/A	N/A
Midline	0/96 (0%)	14/110 (12.7%)	N/A	16/29 (55.2%)
Bilateral	1/96 (1%)	6/188 (3.2%)	N/A	N/A

*AVM* arteriovenous malformation *MMA* middle meningeal artery, *AVF* arteriovenous fistula, *IA* intra-arterial, *WEB* Woven EndoBridge, *ICA* internal carotid artery, *MCA* middle cerebral artery, *ACA* anterior cerebral artery, *AcomA* anterior communicating artery *AICA* anterior inferior cerebellar artery, *VA* vertebral artery, *PCA* posterior cerebral artery, *PcomA* posterior communicating artery, *PICA* posterior inferior cerebellar artery, *BA* basilar artery, *SCA* superior cerebellar artery

#### Endovascular modality for aneurysm treatments

According to data on endovascular modalities, different types of catheters are used in different approaches to manage aneurysms. The Armadillo catheter was used in flow diversion at a high rate (38.3%), followed by coiling (11.5%) and Woven EndoBridge (WEB) (8.5%) (Table 4)**.** Other modalities such as embolization, contour, stent-assisted coiling, and balloon-assisted coiling had lower rates (ranging from 1.0 to 5.2%). With relatively reduced utilization in WEB (9.1%), flow diversion (2.6%), contour (1.5%), and balloon-assisted coiling (1.1%), the RIST catheter showed its maximum proportions in coiling (17.2%), embolization (16.4%), and stent-assisted coiling (14.9%). The most common modality for the BMX 81 catheter was flow diversion (20%), which was followed by embolization (7.5%), WEB (5%), stent-assisted coiling (3.8%), and coiling (2.5%). The Zoom RDL catheter was commonly utilized for flow diversion (17.2%), but less frequently for balloon-assisted coiling (3.4%). No significant differences were observed in the proportions of endovascular modalities utilized between the Armadillo and RIST systems (Table 6).

#### Target vessel

The internal carotid artery (ICA) was the most prevalent target vessel for the Aramdillo and RIST catheters, accounting for 6/9 (66.7%) and 124/272 (45.6%) cases, respectively (Table 4). For the Zoom RDL catheters, on the other hand, the middle cerebral artery (MCA) was the main target, as seen in 18/29 (62.1%) cases. For the BMX 81 catheter, there was insufficient data available to determine its most common target vessel.

#### Radial laterality and laterality of pathology

Right radial access was overwhelmingly preferred across all devices. For instance, the Armadillo, BMX 81 catheter were used exclusively with right radial access (100% of cases) (Table 4). The RIST catheter showed an adoption of left radial access at 7.8% (56/719 cases), followed by Zoom RDL catheter at 3.4% (1/29 cases).Right-sided pathologies were the most common for the Armadillo (56/96, 58.3% and RIST catheters (280/382, 73.3%) (Table 4). Contrastingly, with the Zoom RDL system, midline pathologies were most prevalent (16/29, 55.2%). Again, there was insufficient data for the BMX 81 system to determine its most common laterality of pathology.

### Outcomes

#### Procedural and radiological outcomes

The RIST catheter had the longest relative procedure time (67.1 ± 57.6 min), followed by Armadillo (60.9 ± 31.1 min) (Table 5). Pooled data was insufficient to calculate mean procedure times for the BMX 81 and Zoom RDL catheters. The BMX 81 (37 min), Armadillo (29.2 ± 19.1 min), RIST (27.7 ± 17.7 min), and Zoom RDL (17.1 ± 16.4) catheters followed.

**Table 5:** Summary of outcomes

Variable	Armadillo	RIST	BMX 81	Zoom RDL
Procedure time, mean (SD), min	60.9 (31.1)	67.1 (57.6)	N/A	N/A
Fluoroscopy time, mean (SD), min	29.2 (19.1)	27.7 (17.7)	37	17.1 (16.4)
Raymond Roy occlusion classification				
Grade I	17/33 (51.5%)	34/45 (75.6%)	N/A	N/A
Grade II	10/33 (30.3%)	4/45 (8.9%)	N/A	N/A
Grade III	6/33 (18.2%)	7/45 (15.6%)	N/A	N/A
Failure to catheterize the target vessel	1/162 (0.6%)	15/484 (3.1%)	N/A	3/29 (10.3%)
Complications				
Decreased hand perfusion	1/96 (1%)	0/110 (0%)	N/A	N/A
Artery vasospasm	0/96 (0%)	5/499 (1.0%)	1/40 (2.5%)	N/A
Hematoma	0/96 (0%)	5/284 (1.8%)	N/A	N/A
Neurological complications	3/96 (3%)	20/619 (3.2%)	N/A	2/29 (6.9%)
Transfemoral conversion	2/96 (2%)	22/719 (3.1%)	N/A	2/29 (6.9%)
Procedure-related	4/105 (4%)	20/719 (2.8%)	4/80 (5%)	2/29 (6.9%)

Pooled data on the Raymond Roy Occlusion Criteria, which assesses the degree of aneurysm occlusion, was only available for the Armadillo and RIST catheters. The greatest proportion of aneurysm cases treated with the Armadillo catheter was, followed by Raymond and Roy classification Grade I (complete occlusion) (17/33, 51.5%), and then Grade II (10/33, 30.3%) and Grade III (6/33, 18.2%) (Table 5). For the RIST catheter, the highest proportion of aneurysm cases was in Grade I (complete occlusion) (34/45, 75.6%), followed by Grade III (7/45, 15.6%)), and then Grade II (4/45, 8.9%). The rate of adequate occlusion (grade I and II) was comparable between Armadillo and RIST (81.1% vs 84.5%).

The Armadillo had significantly lower failure to catheterize the target vessel (0.0% vs. 3.04%, *P* = 0.036) than RIST (Table 6). The Zoom RDL catheter had relatively higher failure rate of 10.3% (Table 5). Insufficient data was available to assess the failure rate of the BMX 81 catheter.

**Table 6:** Comparison of armadillo vs. RIST

Variable	Proportion	95% CI	*P*	Tau	H-Statistic	I^2^
Females	69.2% vs. 52.7%	(39.2% – 92.7%) vs. (42.9% – 62.5%)	**0.038**	0.093	1.74	67
Reason for procedure						
Aneurysm treatment	78.9% vs. 26.02%	(0.0% – 100%) vs. (2.9% – 60.75%)	0.09	0.37	3.64	92.5
AVM treatment	4.5% vs. 7.6%	(0.0% – 71.1%) vs. (1.3% – 18.1%)	0.46	0.08	2.2	78.7
MMA embolization	5.25% vs. 13.9%	(0.0% – 90.7%) vs. (11.9% − 16%)	0.072	0.07	1.61	61.4
AVF treatment	8.4% vs. 3.8%	(0.0% – 38.4%) vs. (0.0% – 62.5%)	0.11	0.63	1.64	62.6
Intracranial stent	1.5% vs. 9,1%	(0.0% – 97.5%) vs. (0.0% – 68.7%)	0.35	0.2	4.05	94
Endovascular modality						
Stent-assisted coiling	5.2% vs. 8.3%	(3.3% – 7.5%) vs. (0.0% – 31%)	0.5	0.02	3.7	92.7
Flow diversion	61.8% vs. 25.1%	(0.0% – 100%) vs. (11% – 42.3%)	0.18	0.09	3.24	90.4
Embolization	4.1% vs. 20.3%	(0.0% – 100%) vs. (8.3% – 35.8%)	0.16	0.033	3.74	92.9
WEB	8.4% vs. 10.2%	(0.0% – 38.4%) vs. (0.0% – 35.6%)	0.65	0.0055	1.7	65.4
Failure to catheterize the target vessel	0.0% vs. 3.04%	(0.0% – 2.0%) vs. (0.2% – 8.1%)	**0.036**	0.0038	1.4	49.9
Procedure-related complications	2.4% vs. 2.7%	(0.0% – 45.5%) vs. (0.11% – 7.3%)	0.88	0.0062	1.7	64.6

#### Complications

There were no reported cases of arterial spasm or hemorrhage for the Armadillo catheter, with minimal rates of transfemoral conversion (2%), neurological complications (3%), procedure-related complications (4%), and decreased hand perfusion (1%) (Table 5). With no instances of reduced hand perfusion, the RIST catheter had a 3.2% rate of neurological complications, a 3.1% rate of transfemoral conversion, and a 1.8% rate of hematoma. The BMX 81 catheter had a 2.5% rate of arterial vasospasm and a 5% rate of procedure-related complications. The Zoom RDL catheter had consistent rates of procedure-related, transfemoral conversion, and neurological complications, all at 6.9%, with insufficient data on other complications. There was no significant difference between the Armadillo and RIST catheters in procedure-related complications (Table 5).

### Quality assessment

Quality assessment of included studies using ROBINS-I showed a low and moderate RoB in four and seven studies, respectively.

### DISCUSSION

Although, recently neuroendovascular procedures through radial access gained attention, these procedures were mainly performed using femoral-specific catheters. The Armadillo catheter, due to its softer tip and enhanced flexibility, is highly effective for radial access because it minimizes vascular trauma and allows smooth navigation through arterial pathways [6]. However, due to its flexibility, the Armadillo catheter loses some stability in intricate intracranial interventions. The RIST catheter is made with a stiffer shaft and, therefore, enjoys greater stability and support; it is ideal for complex procedures such as aneurysm repair and flow diversion. This, however, comes at the cost of navigating through challenging vascular geometries. The BMX 81 catheter is compact and robust by design; hence, it is very suitable for large device support in complex embolization or stenting procedures. However, it is less effective in accessing curved or distal vessels because of its limited flexibility [8, 18]. The Zoom RDL catheter combines a tapered distal tip and smooth body, balancing flexibility and support. However, repeated insertion may be associated with tip damage, necessitating device replacement or procedural conversion [6, 9]. The design of the Ballast catheter, with a soft distal segment and stiff proximal shaft, represents a compromise between flexibility and usability for procedures such as aneurysm coiling or stent placement. However, it may occasionally exhibit instability when navigating complex aortic arch anatomy or accessing tortuous supra-aortic vessels, challenges that are not unique to this catheter but have been particularly noted in some reports evaluating its performance [19, 20]. Subtle nuances are underscored; thus, catheter selection has to be appropriate to the anatomy and the procedural requirement for any neuro-endovascular intervention.

Radial-specific catheters are intentionally engineered to address the anatomical constraints of TRA, such as narrower artery caliber and sharp subclavian–aortic angles. These devices incorporate features such as hydrophilic coatings, variable stiffness profiles (e.g., a stiff proximal shaft and a flexible distal segment), and reduced outer diameters to enhance navigation and minimize complications. Notably, FDA and CE approvals are now specifically available for radial neurointerventional use. For example, the RIST catheter features a 29.5 cm hydrophilic distal segment designed for trackability and procedural success in early experience [11]. Similarly, Zoom RDL and BMX 81 offer dedicated engineering tailored for radial proximal support and distal trackability [17]. Conversely, radial-adaptable catheters such as the Ballast were originally developed for transfemoral applications but have gained wide use in radial procedures due to their favorable mechanical characteristics. Studies have reported the Ballast’s balanced stiffness profile and excellent support, making it highly effective for radial navigation [8]. Successful navigation and favorable support of the Ballast catheter in TRA procedures may be related to its cone-shaped design, with a proximal outer diameter of 0.106 inches and a distal outer diameter of 0.100 inches [8]. These findings emphasize that while device engineering for radial access enhances outcomes, successful application also depends on the catheter’s physical characteristics and operator technique, even in devices not originally intended for TRA use. Altogether, the current evidence supports a nuanced view that both dedicated and adaptable devices can perform well depending on procedural demands [4].

This systematic review and meta-analysis indicate large variations in performance, success rates, and complication rates among various radial-specific catheters available for neuroendovascular procedures. Armadillo system showed a 0.0% (95%CI: 0.0% – 2.0%) pooled failure rate in target vessel catheterization, and minimum complication rates such as diminished hand perfusion were noted in only 1% of cases. Conversely, the RIST catheter has presented an increased failure rate of 3.04% with higher complication rate (2.8% vs. 4.3%) than Armadillo. In turn, both the Zoom RDL (10.3%) and Ballast systems (12.1%) showed higher procedural failure rate than Armadillo and RIST. These results underline the performance variation of catheters and indicate the importance of anatomical considerations, both of the patient and the device, in the choice of radial access system in neuro-endovascular interventions [6, 19].

Structural differences between the Armadillo and RIST catheters are pivotal in understanding their clinical applications [6]. The dual-mode design of Armadillo allows an operator to switch between tracking mode and support mode inclusively with adjustments for distal segment stiffness to be made to facilitate the traversal of challenging vascular angles while providing additional stability once in the target position. On the other hand, RIST with, a 5 F diagnostic and a 7 F guide, presents a stiffer proximal part that enables better stability in its advancement, while its distal tip is softer to move smoothly through tortuous vascular pathways [6, 7, 14]. These contrasting structural features reflect distinct approaches toward achieving procedural success: whereas the Armadillo focuses on dynamic adaptability, the RIST is designed with a view to consistent proximal support and ease of navigation. These design elements bear on the subtle differences that are likely seen in procedural outcomes in the form of a greater aneurysm occlusion rate and slightly higher propensity for radial artery vasospasm with the RIST [6, 7]. Catheter size and structural design can substantially affect procedural outcomes. Larger catheters improved the procedural support, enhancing stability in complex cases. Although, they may reduce trackability through tortuous vessels and potentially increase the risk of vascular deformation during procedure. Moreover, specific catheters may be preferred for certain procedures, depending on their design, which can influence the types and rates of complications observed. Therefore, careful selection of catheter type based on both anatomy and procedural goals is important for optimizing outcomes.

El Naamani et al. [6] compared the Armadillo and RIST catheters in neurointervention procedures. They reported a comparable success rate (Armadillo = 1.0% vs. RIST = 0.9%, *P* = 0.18) and complications (Armadillo = 1.0% vs. RIST = 2.8%, *P* = 0.55), emphasizing the high technical feasibility and safety of both devices. Although our findings align with their report, we found a significant lower failure rate in procedures using Armadillo compared with RIST. Abecassis et al. similarly offers a procedural success rate of 96.1% and an overall complication rate of 3.3% for RIST catheter. Interestingly, RIST’s 079 inner diameter, allows using larger 5 F intermediate catheters for better support during complex interventions. Besides, transitions in this catheter have been optimized to minimize the possibility of prolapse and allow stability when tracking along challenging anatomical trajectories, such as the aortic arch and great vessels. These unique features contribute to a lower conversion rate, 1.3%, to femoral access compared with traditional femoral access systems.

#### Future Directions and Limitations

The current study had a few limitations. The retrospective nature of the included studies may limit the generalizability of the findings to broader clinical settings. To address this limitation in future research, it is recommended that standardized protocols for patient selection and data reporting in neuroendovascular procedures be established and implemented universally. Additionally, limited data availability for some catheters, such as the BMX 81 and Zoom RDL systems, restricted our ability to perform direct comparisons across all devices. Although the current study provides insightful results for these devices, more large multicenter studies are required to confirm our findings. Last but not least, most of the included studies were conducted in high-volume centers with experienced operators. This may introduce a bias toward more favorable outcomes for specific devices, necessitating a cautious interpretation of the findings. Future research should incorporate data from a broader range of institutions, including low- and medium-volume centers, to enhance the applicability of results across diverse clinical environments.

## CONCLUSION

The radial-specific catheter showed a reliable and effective tool for neuroendovascular interventions using the trans-radial approach, with consistent results in different clinical settings. Differences in complication rates, however, bring to the forefront the need for critical patient selection, improvement in procedural strategies, and further innovation in catheter engineering. These findings further solidify the surging importance of radial-specific catheters in the radial-first approach and provide promising insights, which still have more room for better outcomes and fewer complications.

## Data Availability

The data is available from the corresponding author on reasonable request.
